# Supplementary material for: New tolerance factor to predict the stability of perovskite oxides and halides
Source: Sci Adv. 2019 Feb 8;5(2):eaav0693. doi: 10.1126/sciadv.aav0693 (PMC6368436; doi:10.1126/sciadv.aav0693)
Supplement: http://advances.sciencemag.org/cgi/content/full/5/2/eaav0693/DC1 [file supp_5_2_eaav0693__index.html]

Science Advances | Science Advances

## Supplementary Materials

**The PDF file includes:**

- Legend for table S1
- Table S2. Confusion matrices for τ (above) and *t* (below).
- Legends for tables S3 and S4
- Fig. S1. Comparing the performance of *t* and τ by composition.
- Fig. S2. Sigmoidal relationship between *P*(τ) and τ.
- Fig. S3. (*t*, μ) structure map for 576 *ABX*3 solids.

Download PDF

**Other Supplementary Material for this manuscript includes the following:**

- Table S1 (.csv format). The 576 *ABX*3 used for training and testing τ.
- Table S3 (.csv format). Additional information associated with Fig. 2D.
- Table S4 (.csv format). Double perovskite oxides and halides.

**Files in this Data Supplement:**

- Adobe PDF - aav0693\_SM.pdf
