# Supplementary material for: New tolerance factor to predict the stability of perovskite oxides and halides
Source: Sci Adv. 2019 Feb 8;5(2):eaav0693. doi: 10.1126/sciadv.aav0693 (PMC6368436; doi:10.1126/sciadv.aav0693)
Supplement: http://advances.sciencemag.org/cgi/content/full/5/2/eaav0693/DC1 [file aav0693_SM.pdf]

Science Advances
AAAS

advances.sciencemag.org/cgi/content/full/5/2/eaav0693/DC1

Supplementary Materials for

New tolerance factor to predict the stability of perovskite oxides and halides

Christopher J. Bartel*, Christopher Sutton, Bryan R. Goldsmith, Runhai Ouyang, Charles B. Musgrave, Luca M. Ghiringhelli*, Matthias Scheffler

*Corresponding author. Email: ghiringhelli@fhi-berlin.mpg.de (L.M.G.); christopher.bartel@colorado.edu (C.J.B.)

Published 8 February 2019, *Sci. Adv.* **5**, eaav0693 (2019)
DOI: 10.1126/sciadv.aav0693

The PDF file includes:

Legend for table S1
Table S2. Confusion matrices for τ (above) and *t* (below).
Legends for tables S3 and S4
Fig. S1. Comparing the performance of *t* and τ by composition.
Fig. S2. Sigmoidal relationship between *P*(τ) and τ.
Fig. S3. (*t*, μ) structure map for 576 ABX_3 solids.

Other Supplementary Material for this manuscript includes the following:

(available at advances.sciencemag.org/cgi/content/full/5/2/eaav0693/DC1)

Table S1 (.csv format). The 576 ABX_3 used for training and testing τ.
Table S3 (.csv format). Additional information associated with Fig. 2D.
Table S4 (.csv format). Double perovskite oxides and halides.

Supplementary Materials

Table S1. The 576 ABX_3 used for training and testing τ. Within this table – *exp label* = 1 corresponds with experimentally labeled perovskites and *exp label* = –1, nonperovskites; *is train* = 1 corresponds with a training set compound and *is train* = –1 a test set compound; τ is provided as *tau*, classification using τ as *tau pred*, classification using *t* as *t pred*, and τ-derived probabilities, P(τ) as *tau prob*. *A, B, X,* n_A, n_B, n_X, r_A, r_B, r_X, and *t* are also provided and named as they are in the text.

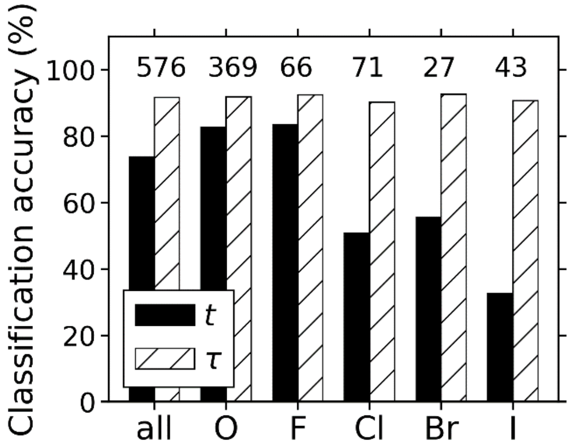

Fig. S1. Comparing the performance of *t* and τ by composition. Classification accuracy on the full set of 576 experimentally characterized ABX_3 solids (*all*) and by compounds containing $X = O^-$, F^-, Cl^-, Br^-, I^-. The number appearing above each pair of columns corresponds with the number of compounds evaluated within each set.

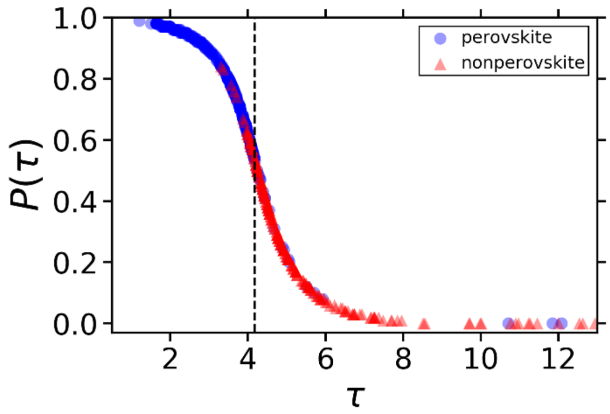

Fig. S2. Sigmoidal relationship between *P*(τ) and τ. A comparison of Platt-scaled classification probabilities, $P(\tau)$, versus τ for the 576 experimentally characterized ABX_3 solids. The outlying compounds at $\tau > 10$ that are labeled perovskite yet have small $P(\tau)$ are $PuVO_3$, $AmVO_3$, and $PuCrO_3$, which may indicate poorly defined radii or experimental characterization.

Table S2. Confusion matrices for τ (above) and *t* (below). N = number of samples; TP = true positives (experimentally realized perovskites which are predicted perovskite); FP = false positives (predicted perovskites which are not experimentally realized perovskites); TN = true negatives (predicted nonperovskites which are experimentally realized nonperovskites); FN = false negatives (predicted nonperovskites which are experimentally realized perovskites); precision = TP/(TP+FP); recall = TP/(TP+FN); F1 score = (2*precision*recall)/(precision + recall); kappa = Cohen's Kappa score; accuracy = (TP+TN)/N.

tau:

anion	N	TP	FP	TN	FN	precision	recall	F1 score	kappa	accuracy
all	576	293	28	235	20	0.913	0.936	0.924	0.832	0.917
O	369	217	15	122	15	0.935	0.935	0.935	0.826	0.919
F	66	50	4	11	1	0.926	0.980	0.952	0.768	0.924
Cl	71	18	7	46	0	0.720	1.000	0.837	0.769	0.901
Br	27	5	2	20	0	0.714	1.000	0.833	0.787	0.926
I	43	3	0	36	4	1.000	0.429	0.600	0.557	0.907

t:

anion	N	TP	FP	TN	FN	precision	recall	F1 score	kappa	accuracy
all	576	295	133	130	18	0.689	0.942	0.796	0.453	0.738
O	369	216	48	89	16	0.818	0.931	0.871	0.610	0.827
F	66	49	9	6	2	0.845	0.961	0.899	0.432	0.833
Cl	71	18	35	18	0	0.340	1.000	0.507	0.207	0.507
Br	27	5	12	10	0	0.294	1.000	0.455	0.236	0.556
I	43	7	29	7	0	0.194	1.000	0.326	0.073	0.326

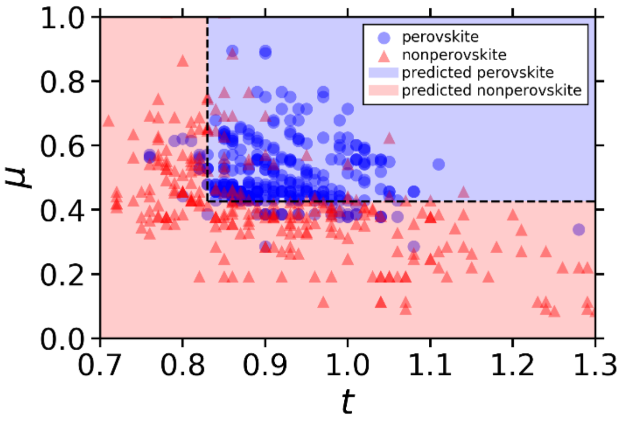

Fig. S3. (t, μ) structure map for 576 ABX_3 solids. A decision tree trained on all compounds finds the optimal bounds for perovskites stability to occur for $t > 0.830$ and $\mu > 0.426$, which results in a classification accuracy of 85%.

Table S3. Additional information associated with Fig. 2D. Columns are named as described for table S1 with the additional columns: *source* – corresponding with the DOI from which the DFT decomposition enthalpy was obtained and *dHdec (meV/atom)* – the PBE-computed decomposition enthalpy of the cubic structure.

Table S4. Double perovskite oxides and halides. All charge-balanced compounds described in the context of **Fig. 3** are provided. Columns are named as described for **table S1** and **table S3**. An additional column, *icsd label*, provides the assignment of perovskite/nonperovskite extracted from the ICSD if available.
